# The function of tcf3 in medaka embryos: efficient knockdown with pePNAs

**DOI:** 10.1186/s12896-017-0411-0

**Published:** 2018-01-09

**Authors:** Gerlinde Doenz, Sebastian Dorn, Narges Aghaallaei, Baubak Bajoghli, Elisabeth Riegel, Michaela Aigner, Holger Bock, Birgit Werner, Thomas Lindhorst, Thomas Czerny

**Affiliations:** 1 Department for Applied Life Sciences, University of Applied Sciences, FH Campus Wien, Helmut-Qualtinger-Gasse 2, A-1030 Vienna, Austria; 2 Centre for Organismal Studies (COS), University of Heidelberg, Im Neuenheimer Feld 230, 69120 Heidelberg, Germany; 3 Department of Hematology, Oncology, Immunology, Rheumatology and Pulmonology, University Hospital Tübingen, Otfried-Mueller-Strasse 10, 72076 Tübingen, Germany; 4 Sandoz GmbH, Biochemiestraße 10, A-6250 Kundl, Austria; 5 CAST Gründungszentrum GmbH, Wilhelm-Greil-Straße 15, A-6020 Innsbruck, Austria; 6 UGISense AG, c/o Nordwind Capital GmbH, Residenzstrasse 18, 80333 München, Germany

**Keywords:** PNA, Knockdown, Medaka, Tcf3, Groucho, Tle

## Abstract

**Background:**

The application of antisense molecules, such as morpholino oligonucleotides, is an efficient method of gene inactivation in vivo. We recently introduced phosphonic ester modified peptide nucleic acids (PNA) for in vivo loss-of-function experiments in medaka embryos. Here we tested novel modifications of the PNA backbone to knockdown the medaka *tcf3* gene.

**Results:**

A single *tcf3* gene exists in the medaka genome and its inactivation strongly affected eye development of the embryos, leading to size reduction and anophthalmia in severe cases. The function of Tcf3 strongly depends on co-repressor interactions. We found interactions with Groucho/Tle proteins to be most important for eye development. Using a dominant negative approach for combined inactivation of all *groucho/tle* genes also resulted in eye phenotypes, as did interference with three individual *tle* genes.

**Conclusions:**

Our results show that side chain modified PNAs come close to the knockdown efficiency of morpholino oligonucleotides in vivo. A single medaka *tcf3* gene combines the function of the two zebrafish paralogs *hdl* and *tcf3b*. In combination with Groucho/Tle corepressor proteins Tcf3 acts in anterior development and is critical for eye formation.

**Electronic supplementary material:**

The online version of this article (10.1186/s12896-017-0411-0) contains supplementary material, which is available to authorized users.

## Background

Antisense molecules which inhibit mRNA activity through a Watson-Crick base pair mechanism have been studied for several decades. Blocker molecules, such as morpholino oligonucleotides and peptide nucleic acids (PNAs), show high specificity and low toxic effects (reviewed by Summerton [1]). The morpholino backbone consists of 6-membered morpholine rings connected by non-ionic phosphorodiamidate linker-units [2]. In PNAs the phosphate ribose ring of DNA is replaced with repeating N-[2-aminoethyl]-glycine (aeg) units linked by amide bonds. This provides a neutral backbone and results in complementary PNA/DNA strands having stronger binding affinity than DNA/DNA [3]. In addition, introduction of mismatches destabilizes the PNA/DNA duplex more than they do in DNA/DNA. PNAs are highly stable against enzymatic degradation and changes in temperature and pH. Moreover, due to their short length (13-18 bases) the likelihood that secondary structures form is reduced which results in stable and sequence specific probes [4–6]. Unmodified aegPNAs, however, show low efficiency in vivo, therefore, various terminal- and internal modifications have been tested. For example, the addition of lysine residues improves their solubility [7]. The introduction of negative charges in mixed sequence HypNA-PNAs allowed specific down regulation of target genes in zebrafish embryos [8]. Recently, pePNAs which contain phosphonic ester (pe) side chains in an otherwise non-modified aegPNA backbone were successfully applied to target *six3* in medaka embryos. In particular combined versions containing both aeg non-modified- and pe-modified PNA blocks were most effective. In a direct comparison morpholino oligonucleotides were active at lower concentrations, but also showed higher toxicity [9].

Tcf/Lef (T-cell factor/lymphoid enhancer factor) proteins belong to the high mobility group (HMG) box-containing family of transcription factors [10]. They are the most distal players of the canonical Wnt pathway and the main partner for β-catenin in gene regulation [11, 12]. In the absence of Wnt ligands, β-catenin is degraded in the cytosol and Tcf/Lef interacts with transcriptional co-repressors such as Groucho/Tle (Groucho/Transducin-like enhancer) and CtBP (C-terminus Binding Protein), thereby repressing target gene expression [13]. Upon Wnt stimulation, β-catenin is stabilized and translocated into the nucleus. Nuclear accumulation leads to interaction of β-catenin with Tcf/Lef which promotes specific gene expression [11].

In non-vertebrates transcriptional activation and repression is regulated solely by a single *tcf* gene, for example *pangolin* in *Drosophila* and *POP-1* in *C. elegans*, whereas vertebrates have four, more specialized and partly redundant homologues (*tcf1, tcf3, tcf4* and *lef1*) [11]. While Tcf1 and Tcf4 proteins play a role in both activation and repression, Lef1 primarily functions as an activator [14] and Tcf3 appears to function exclusively as a repressor [15]. In mice, *tcf3* knockout experiments resulted in gastrulation defects that resemble ectopic Wnt expression such as duplication of the anterior-posterior (AP) axis. At later stages abundant neuroectodermal cells and defective neural patterning were observed [16]. In addition, Tcf3 seems to be a regulator of pluripotency. Embryonic stem (ES) cells show high levels of Tcf3 and only low Wnt pathway activity, thereby remaining in a balanced state between pluripotency and differentiation. When *tcf3* is knocked down or *Wnt* genes are overexpressed, the ES cells do not undergo efficient differentiation and the balance is tipped towards pluripotency [17, 18]. In zebrafish two *tcf3* genes have been reported, *headless hdl*/*tcf3* [19] and *tcf3b* [20], which both act as repressors. At the shield stage (mid-gastrulation) *hdl* is broadly expressed in the epiblast while *tcf3b* demonstrates only weak expression, at late gastrulation both genes are expressed in the rostral neuroectoderm and later on throughout the brain with a gap of expression in the mid-hindbrain boundary (MHB) [20]. In *hdl* mutants the forebrain is lost while the expression of genes that characterize the MHB is expanded [19]. This phenotype can be rescued by *tcf3b* overexpression, indicating redundant functions of *hdl* and *tcf3b* [20]. *tcf3b* knockdown results in smaller heads but otherwise normal brain patterning, whereas interference with both *tcf3* genes leads to strong caudalization [20].

One mechanism of Tcf3-mediated repression results from its interaction with Groucho/Tle proteins [13, 21, 22], which are transcriptional co-repressors that lack direct DNA-binding ability. Instead they bind to DNA-bound proteins and assist in repression (reviewed by Turki-Judeh and Courey [23]). In the case of Tcf3, Groucho/Tle tetramers bind to an N-terminally located groucho interaction domain [24]. This interaction is disrupted by the Groucho/Tle repressor Aes [21], which is a truncated form of Groucho/Tle consisting only of the N-terminal Q- and GP-domains [25, 26].

Here we analyzed the function of *tcf3* in medaka embryos using both gain- and loss-of-function experiments. For the knockdown we used pePNAs and morpholino oligonucleotides. Novel side chain modifications further improved the activity of the PNAs, resulting in knockdown efficiencies directly comparable to those of the morpholino oligonucleotides. Furthermore, we analyzed the effect of Groucho/Tle proteins on the repressive function of Tcf3 using a dominant negative approach and antisense oligonucleotides.

## Results

### Isolation and expression of the medaka tcf3 gene

**Fig. 1:**
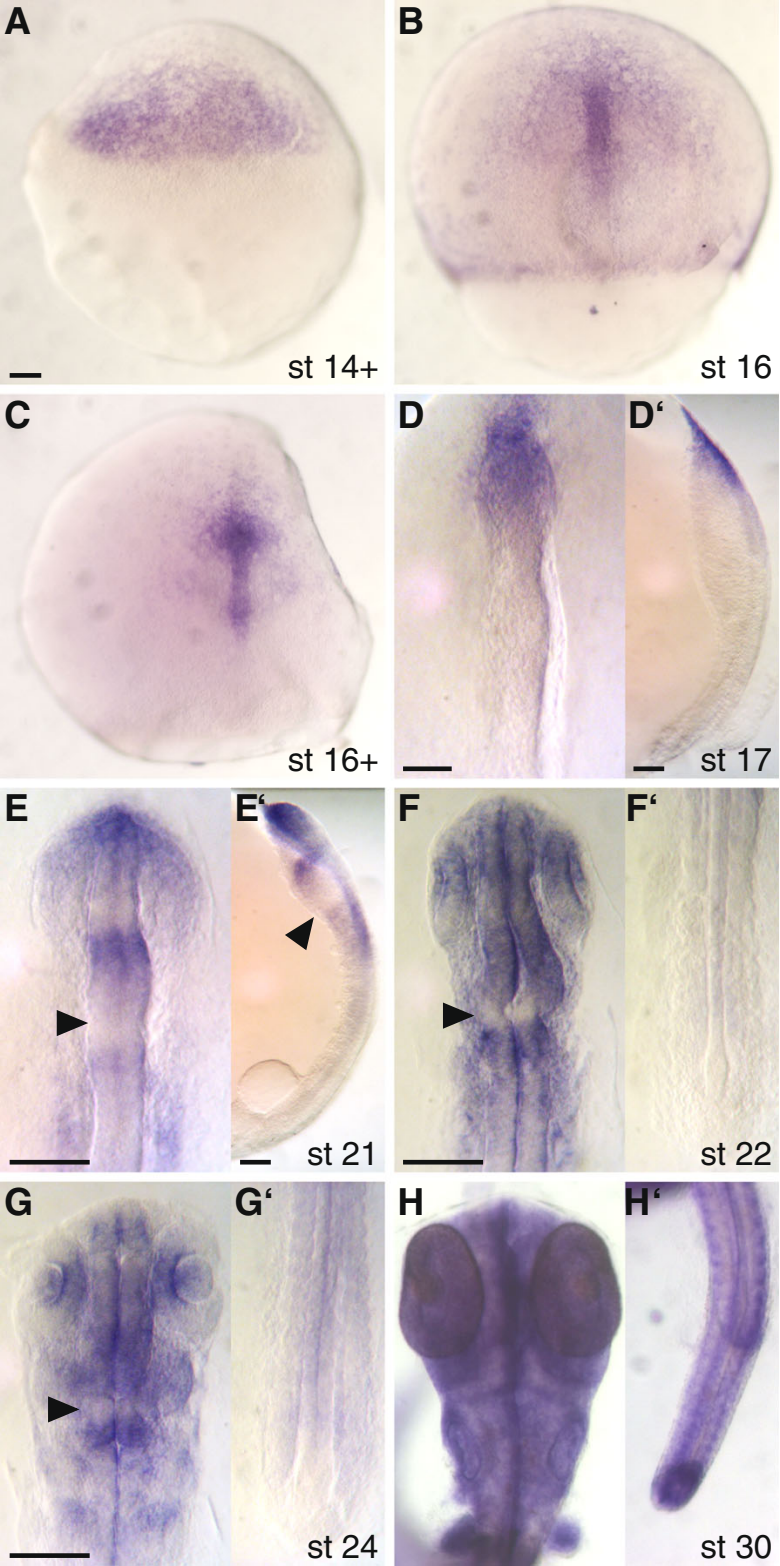
*tcf3* expression in medaka embryonic development. Whole mount in situ hybridization experiments were performed in wild type embryos at the indicated stages, using a digoxigenin-labelled RNA probe against *tcf3*. Embryos are shown in dorsal view, anterior at the top. (**f-h**) Flat mounts; (**f'**-**h'**) tail view. During gastrulation (**a**-**c**) *tcf3* is expressed throughout the epiblast and the embryonic body (**c**-**d**). In neurula the expression becomes restricted to the head (**d,d'**) from where it spreads caudally and divides into a forebrain, midbrain, and hindbrain section (**e**;**e'**). A gap in expression at the mid-hindbrain boundary (**f**-**h**, arrowhead) is closed at later stages (**h**). In later stages, expression is observed throughout the entire body (**g**-**h'**). Scale bars 100 μm; **a**, **a**-**c**; **f**, **f**-**f′**; **g**, **g**-**h′**. Abbreviations: st, stage

We used a BLAST search to identify homologs of the *tcf3* gene in the medaka genome, using the mouse Tcf3 protein sequence as a template. In this work, we identified several homologs of *tcf/lef* genes, however, only one single hit matched the *tcf3* gene (corresponding Ensembl transcript ENSORLT00000014810 located on chromosome 9 and encoding for a 600 amino acid protein). The presence of a single *tcf3* gene in medaka was also reported previously [27]. In order to study the expression pattern of the medaka *tcf3* gene, we isolated a cDNA fragment by RT-PCR and performed whole mount in situ hybridization (Fig. 1). During gastrulation, *tcf3* is broadly expressed throughout the embryonic shield (40% epiboly; Fig. 1a), followed by an anterior shift towards the prospective head region (Fig. 1c) and into the embryonic body (Fig. 1b,c) at late gastrula stage. At the neurula stage the expression becomes restricted to the head region (Fig. 1d,d’) and appears in posterior regions of the embryo later during somitogenesis (Fig. 1e-h’). When the first subdivisions of the brain can be distinguished, *tcf3* expression becomes segmented into the telencephalon, midbrain and hindbrain domains (Fig. 1e,e’). Similar to the expression of zebrafish *tcf3* [20], medaka *tcf3* is not expressed in the MHB (Fig. 1e-g). Furthermore, *tcf3* is also expressed in the eyes and the otic placodes (Fig. 1e). During somitogenesis, *tcf3* expression further extends caudally (Fig. 1f-h’), where it is then found in the pectoral fins and the somites in late stages (Fig. 1h, h’). Interestingly, the expression pattern of *tcf3* in medaka combines the expression domains of the zebrafish genes *hdl (tcf3a)* and *tcf3b* [20].

### The medaka tcf3 gene is crucial for eye development

**Fig. 2:**
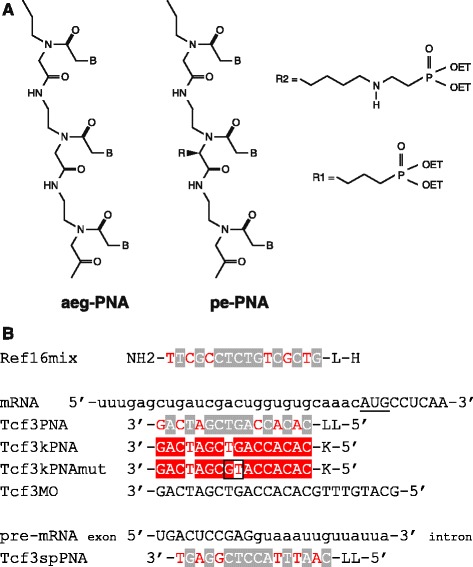
Sequences of medaka *tcf3* antisense molecules and their targets. **a** Schematic chemical structure of aegPNAs, pePNAs. Mixed PNAs contain C3- (R1) or lysine-phosphonic-ester- (R2) residues. **b** mRNA targets are shown in the 5′-3′ orientation, capital letters indicate the coding region. The AUG start codon is underlined. Pre-mRNA represents the unspliced mRNA precursor of the medaka *tcf3* gene with exon 1 sequences shown in capital letters (exon 1 includes the first 62 amino acids of Tcf3). Tcf3MO represents a morpholino oligonucleotide, all other sequences PNAs. aegPNA components are shown with grey overlay, pePNA components with C3-phosphonic-ester (R1) side chains are shown in red letters, those with lysine-phosphonic-ester (R2) side chains in red overlay. Boxed bases represent mismatches. All PNA and morpholino oligonucleotides are shown in 3′-5′ orientation. L represents a trimethyl-lysine and K a dimethyl-lysine

**Fig. 3:**
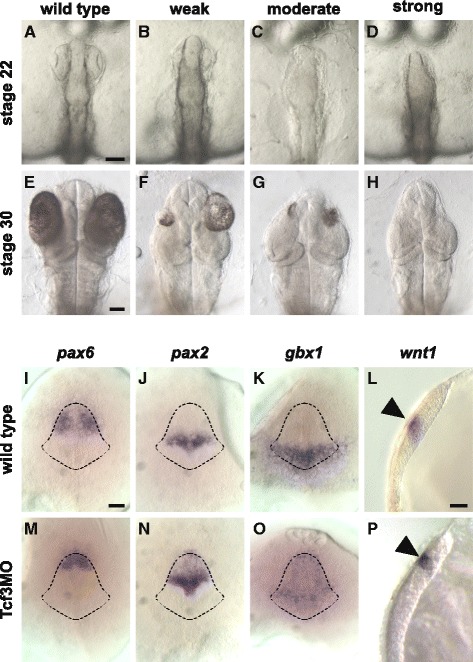
Loss of function phenotypes for the *tcf3* gene in medaka. Embryos at the 1-cell stage were co-injected with 300 μM morpholino oligonucleotide (Tcf3MO) and 1 μg/mL FITC-dextran. The pairs **b** and **f**, **c** and **g**, **d** and **h** each show the same embryo at different stages (stage 22, **a**-**d**; stage 30, **e**-**h**). Control embryos (**a**,**e**) were co-injected with 1× Yamamoto’s and FITC-dextran. Pictures of embryos with a fluorescent signal were taken at the indicated stages. Classification was performed after the onset of eye pigmentation, according to the size of the eye: (**b**,**f**) weak, slightly reduced size; (**c**,**g**) moderate, severe size reduction; (**d**,**h**) strong, eye-less. Embryos at stage 16 (**i**-**k**, **n**-**o**) are shown in dorsal view, anterior to the top; except for (**l**,**p**) which are at stage 17 and shown in lateral view. For genotypic analysis (**i**-**p**), whole mount in situ hybridization experiments were performed on MO injected embryos and un-injected wild type controls at stage 16 using digoxigenin-labelled RNA probes against *pax6* (**i**,**m**), *pax2* (**j**,**n**), *gbx1* (**k**,**o**), and *wnt1* (**l**,**p**) at stage 17. (**i**-**k**, **m**-**o**) Broken lines indicate the outlines of the prospective neural domain estimated from the merged expression patterns formed by *pax6*, *pax2* and *gbx1* in wild type embryos (**i**-**k**). An anterior shift of the expression domains was observed for all four genes. (**l**,**p**; arrowhead) *wnt1* expression in the mid-hindbrain boundary. Scale bars 100 μm; **a**, **a**-**h**; **i**, **i**-**k** and **m**-**o**; **l**, **l** and **p**. Abbreviations: MO, morpholino oligonucleotide

In order to understand the function of the medaka *tcf3* gene during embryogenesis, we performed knockdown experiments using PNAs and morpholino oligonucleotides. We used a 16mer mixed pePNA (Fig. 2a) and a 25mer morpholino oligonucleotide sequence directed against the medaka *tcf3* mRNA directly upstream of the translation start codon (Fig. 2b). Injection of PNAs and morpholino oligonucleotides resulted in the same phenotypes. The embryos progressed through gastrulation without apparent defects; however, eye development was later specifically impaired (Fig. 3b-d). For phenotype classification, we separated the embryos into three groups (weak, moderate and strong): weak embryos (Fig. 3b,f) exhibited one or two slightly smaller eyes than the control group, whereas in embryos with a moderate phenotype the size reduction was severe (Fig. 3c,g). Embryos without eyes were categorized as having a strong phenotype (Fig. 3d,h). Interestingly, in zebrafish the phenotypes for the two *tcf3* genes differ considerably, however, in *hdl/tcf3b* double morphants they add up to one combined phenotype representing the complete absence of Tcf3 proteins [20]. Medaka embryos with a strong phenotype correspond to this extreme case, whereas embryos of the weak and moderate groups demonstrate phenotypes in between those for *hdl* [19] and *tcf3b* [20] in zebrafish.

To further characterize the *tcf3* loss-of-function phenotype, we next examined the expression of *pax2*, *pax6*, *gbx1* and *wnt1* (Fig. 3i-p), which is known to be affected by Tcf3 activity [20]. In those embryos the expression domains of all three genes were shifted anteriorly (Fig. 3m-o) compared to the wild type control embryos (Fig. 3i-k). In 48% of the embryos (*n* = 61) the expression domain of the forebrain marker *pax6* was smaller (Fig. 3m) and instead of two separate domains on either side of the prospective head (Fig. 3i), only a single cap-like structure was observed at the most anterior region of the prospective neural domain (Fig. 3m). However, we did not see a complete loss of anterior *pax6* expression. The expression of *pax2*, a marker of the MHB, was shifted anteriorly in 56% of the morphants (*n* = 36), partially overlapping with the *pax6* domain (Fig. 3n) which is in good agreement with the expression pattern found in zebrafish *hdl* mutants [20]. In 37% of the embryos (*n* = 43) an altered expression of the hindbrain marker *gbx1* was observed, where again the entire expression domain was shifted anteriorly (Fig. 3o). In addition, the lateral edges of less severely caudalized embryos pointed anteriorly and in more severe phenotypes, the expression expanded into an arc-like shape, thereby overlapping with the expression domains of both *pax6* and *pax2*. We also analysed the MHB marker *wnt1* in *tcf3* knockdown embryos and found expression in a more anterior position (Fig. 3b). Taken together, the expression of all marker genes clearly indicates the expected anterior shift for *tcf3* inactivation.

### Improvement of PNA specificity

**Fig. 4:**
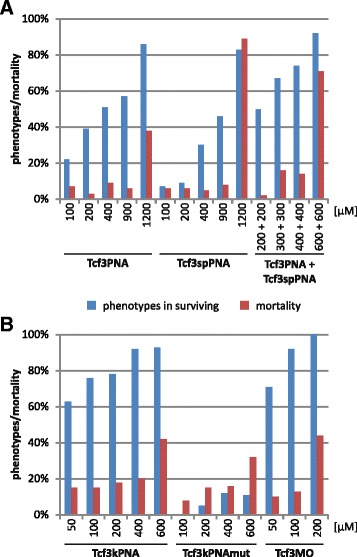
Knockdown efficiencies using modified PNAs. Quantitative evaluation of *tcf3* knockdown experiments. Results for injection of the indicated antisense molecules at the indicated concentrations. The blue bars represent the percentage of embryos with phenotypes among the surviving embryos, the red bars represents the mortality rate (see also Additional file 1)

Qualification of the pigmented retinal structures of the eyes allowed a simple but reliable phenotype classification. Based on these results we could compare the knockdown efficiency of the different antisense molecules. We first used a mixed pePNA (Tcf3PNA), which contains two tri-methyl-lysine (TML) terminal groups. For each concentration the number of phenotypes of the different groups was determined and the total number of *tcf3* knockdown phenotypes among the surviving embryos was calculated (Fig. 4a and Additional file 1). Between 100 μM and 400 μM of Tcf3PNA the total number of phenotypes increased from 22% to 51%. At 600 μM and 900 μM the overall frequency of phenotypes (both 57%) and also the number of dead embryos (6% and 7%, respectively) remained unchanged, which indicates a low toxicity. At 1200 μM the percentage of *tcf3*-specific phenotypes increased to 86%, however, the mortality rate also increased to 38%, indicating elevated toxicity of the PNA at higher concentrations. As expected, these increasing numbers of phenotypes also resulted in a continuous shift from preferentially weak phenotypes at low concentrations to a high proportion of strong phenotypes at high concentrations (Additional file 1). The morpholino oligonucleotide showed phenotypes indistinguishable from the pePNAs, but differed in their efficiency. High rates of *tcf3*-specific phenotypes, 71% and 92%, already appeared at low concentrations, 50 μM and 100 μM, respectively. However, at 200 μM the mortality rate jumped to 44%, indicating toxic effects (Fig. 4b and Additional file 1). At concentrations with high mortality rates, some embryos with unspecific phenotypes appeared which looked similar for both pePNAs and morpholino oligonucleotides. We previously described such unspecifically affected medaka embryos, which have smaller eyes but show severe defects over the entire brain [9]. Such unspecific phenotypes were not detected at non-toxic concentrations. Taken together, the morpholino oligonucleotide was more efficient than the pePNA at low concentrations, but also showed higher toxicity.

Thereafter, we tested the pePNAs’ splice blocking efficiency. A sequence overlapping with both exon 1 and intron 1 was selected and also synthesized as a mixed PNA with a double TML end group (Tcf3spPNA; Fig. 2b). The result of this splice block is a truncated and therefore non-functional protein. Upon PNA injection the same phenotypes were observed as were found for Tcf3PNA, however, with lower efficiency (Fig. 4a and Additional file 1). The highest number of specifically affected embryos (49%) was observed at a concentration of 900 μM. At 1200 μM toxicity became dominant, similar to that seen for Tcf3PNA. We reasoned that a combination of both the translational blocking and the splice blocking PNAs might result in an improved knockdown efficiency. Indeed, co-injection of Tcf3PNA and Tcf3spPNA, each at a concentration of 200 μM, resulted in 50% of the embryos showing phenotypes (Fig. 4a and Additional file 1), which is higher than the percentage of affected embryos for both 400 μM Tcf3PNA (39%) and 400 μM Tcf3spPNA (30%). Therefore, the combined injection of both PNAs led to a synergistic improvement, which was also observed at higher concentrations. At 900 μM (450 μM each) 74% of the embryos showed *tcf3*-specific phenotypes without toxic side effects (those first appeared at 1200 μM).

We then tested lysine-phosphonic-ester (lpe) side chains as a novel backbone modification of the PNAs (Fig. 2a). A mixed 16mer PNA with the same target sequence as Tcf3PNA was synthesized (Tcf3kPNA; Fig. 2b). Injection of these new PNAs resulted in identical *tcf3* specific phenotypes, but in considerably higher numbers (Fig. 4b and Additional file 1). At 100 μM the Tcf3kPNA produced 76% *tcf3* specific phenotypes, compared to 22% for the Tcf3PNA. Similar improvements were also seen for other concentrations. A peak level of 92% phenotypes was observed at 400 μM with the first toxic effects of these modified PNAs appearing at 600 μM. Therefore, the lpe-side chain modification strongly improved the antisense efficiency of PNAs. To test specificity, a two nucleotide substitution was introduced (GT to TG at positions 8 and 9 of the PNA; Tcf3kPNAmut Fig. 2b). This substitution resulted in a dramatic loss of the *tcf3* knockdown phenotypes (Fig. 4b and Additional file 1). At 100 μM none of the embryos showed *tcf3*-specific phenotypes and at 600 μM only 11% did (compared to 93% for the non-mutated version). Therefore, a highly specific antisense function of the K-modified PNAs in an in vivo environment could be observed at low concentrations, directly comparable to the results for morpholino oligonucleotides. Taken together, the repeated appearance of the same phenotypes with various different antisense molecules clearly demonstrated specificity of the *tcf3* knockdown experiments.

### Tcf3 function in eye development is mediated by Groucho/Tle interaction

In order to understand the effect of Tcf3 misexpression during embryogenesis, we injected a heat-inducible *tcf3* construct into the blastomere of one-cell stage wild-type embryos. The heat-inducible promoter allowed us a stage-dependent gain-of-function analysis [28]. We first performed heat treatment at mid-gastrulation (stage 14/15) and analyzed the injected embryos after the onset of eye pigmentation at stage 30. In this context, 31% (*n* = 27) of the injected embryos showed reduced eye size and 8% lacked eyes; neither varying DNA concentration nor heat-induction time altered the eye phenotypes. Hence, based on our results gain-of-function and loss-of-function experiments for *tcf3* resulted in similar phenotypes.

**Fig. 5:**
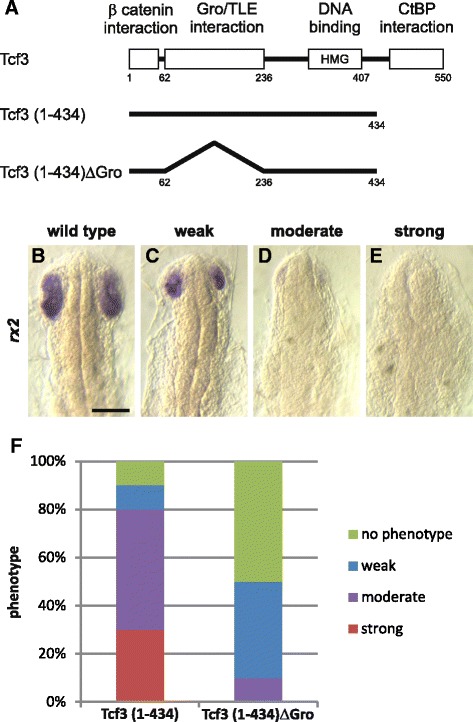
*tcf3* gain of function phenotypes. **a** Schematic representation of full length Tcf3, Tcf3 lacking the CtBP binding site (Tcf3[1-434]), and a C-terminally truncated Tcf3 lacking the Groucho/Tle interaction domain (Tcf3[1-434]ΔGro). **b**-**f** Embryos at the 1-cell stage were injected with either heat-inducible truncated Tcf3 (Gfp:HSE:Tcf3(1-434); 40 ng/μl) or Tcf3 lacking the Groucho/Tle interaction domain (Gfp:HSE:Tcf3[1-434]ΔGro; 40 ng/μl). Heat induction was performed at stage 14-15. Gfp positive embryos were selected at stage 22 for subsequent whole mount in situ hybridization experiments using digoxigenin-labelled RNA probes against *rx2* (**c**-**f**). **b**-**e** Flat mounts are shown in dorsal view, anterior at the top. Phenotypes were categorized according to their eye size and the expression intensity compared to the control: (**c**) weak, small eyes, normal expression intensity; (**d**) small eyes, weak expression; (**e**), missing eyes and expression. **b** Heat treated wild type control. **f** Quantitative results of the injections using *rx2* in situ hybridization. Scale bar 100 μm

We subsequently focused on the functional domains of Tcf3 which mediate transcriptional repression. More precisely, the Tcf3 protein contains two repression domains: a CtBP interaction domain at the C-terminus [29] and a Groucho/Tle interaction domain at the N-terminus [24] (Fig. 5a). We therefore constructed differently truncated *tcf3* cDNAs that either lack the CtBP interaction domain (Tcf3 (1-434)), or both the CtBP and the Groucho/Tle interaction domain (Tcf3 (1-434)ΔGro). Injection of Tcf3 (1-434) cDNA lacking the CtBP binding site, increased the frequency of embryos showing eye phenotypes to 26% (Fig. 5f) compared to 8% for the full-length Tcf3 construct. This indicates that CtBP interaction does not increase eye phenotypes, but instead reduces them. On the other hand, injection of the truncated *tcf3* cDNA lacking both the CtBP- and Groucho/Tle interaction domains (Tcf3 (1-434)ΔGro), resulted in a significantly reduced frequency of embryos with strong eye phenotypes (5%), which suggests that the Groucho/Tle interaction of Tcf3 is more important for eye development than that with CtBP is.

Additionally, we examined the expression of the retina-specific homeobox gene *rx2* in the injected embryos an early stage of eye development [30, 31]. In agreement with the eye phenotypes, three different expression patterns of *rx2* were observed in *tcf3*-injected embryos at stage 21 (Fig. 5b-e). We compared the expression pattern of *rx2* in embryos injected either with Tcf3 (1-434) lacking the CtBP binding site or Tcf3 (1-434)ΔGro lacking both the CtBP and the Groucho/Tle interaction domains. The expression of *rx2* was less reduced in embryos injected with the Tcf3 construct that lacks the Groucho/Tle interaction domain but still retains the DNA binding- and the ß-catenin interaction domains (Fig. 5f and Additional file 2). In particular, misexpressing this truncated version significantly reduced the appearance of eye phenotypes up to 50% in the injected embryos. Our data therefore suggest that Groucho/Tle co-repressor proteins make a crucial contribution to eye development. Moreover, in addition to eye phenotypes, 21% (*n* = 66) of Tcf3(1-434)ΔGro injected embryos exhibited ectopic otic vesicles (Additional file 2B,C). We observed ectopic otic vesicles in the trunk and tail regions of the developing embryos, hence, posterior to the endogenous vesicles (Additional file 2C). Taken together, our results suggest that Tcf3 function in eye development is modulated by the Groucho/Tle corepressor interaction.

### Two Groucho/Tle genes, tle1 and tle2b, have redundant functions in eye development

**Fig. 6:**
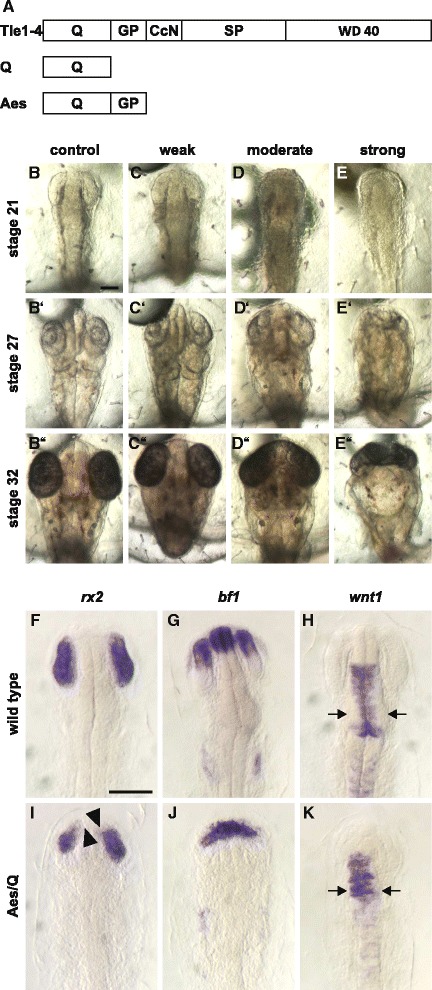
Aes-mediated Groucho/Tle loss of function phenotypes. **a** Schematized presentation of full length Tle protein, the Q-domain, and Aes. For transgenic lines, embryos at the 1-cell stage were injected with 20 ng/μl of DNA (Gfp:HSE:Aes and Gfp:HSE:Q). The F1 generation was heat-induced (10 min, 43.5 °C) at stage 15/16. **b**-**k** Embryos are shown in dorsal view, anterior to the top. **f**-**k** Flat mounts. **c**-**d** Phenotypes of the Aes/Q-mediated Groucho/Tle loss of function were categorized according to their eye phenotype into: (**c**-**c**″) weak, smaller eyes tilted towards the midline; (**d**-**d**”) moderate, beginning cyclopia anterior; strong (**e**-**e**”), cyclopic eye. For whole mount in situ hybridization experiments (**f**-**k**), using the indicated digoxigenin-labelled RNA probes, fixation was performed at stage 21; heat treatment occurred as described before. **i** *rx2* expression indicates a reduction in eye size, the eyes are tilted at the anterior end towards the midline (arrowheads); (**j**) *Bf1* expression is reduced; (**k**) *Wnt1* expression indicates a reduction in midbrain size. Instead of a defined expression close to the midline (**h**, arrows), Aes misexpression resulted in an indistinct expression pattern throughout the midbrain (K, arrows). (**b**-**b**″, **f**-**h**) control embryos were heat-treated wild type embryos. Scale bars 100 μm; **b**, **b**-**e**”; **f**, **f**-**k**

Groucho/Tle co-repressor proteins have been implicated in eye development [32, 33]. Particularly Tle1 and Aes were shown to interact with the transcription factors Six3 and Six6 during early eye development [33]. However, several *groucho/tle* genes are broadly expressed in various developing tissues [34]. Thus, in order to interfere with the function of all Groucho/Tle proteins within one experiment we selected a dominant negative strategy and used a truncated form of Tle4 that contains only the Q domain. The Tle-Q domain is sufficient for interaction with Lef/Tcf proteins and forms a tetrameter upon binding to Lef1 [22]. The Aes protein, which is a truncated member of the Groucho/Tle family, contains only the N-terminal Q and GP domains (Fig. 6a). It has been used repeatedly as a dominant negative form for Groucho/Tle proteins [24, 35] forming intra- as well as intermolecular interactions with other Groucho/Tle proteins via its Q-domain. The resulting tetramers therefore will partially lack the C-terminal domains, which are not present in the Aes protein. Most important at the C-terminus are the domains of WD40 repeats which represent a major protein-protein interaction domain. Inefficient complex formation is therefore the consequence of Aes expression. However, a major criticism of this strategy is that important interactions also originate from the Q-domain. Compared to other Groucho/Tle family members, the Aes Q-domain shows a number of deviations in its amino acid sequence. It is therefore not clear how efficiently the Aes Q-domain can contribute to the interactions in a mixed protein complex. To address this issue, we tested the effect of Aes overexpression on an established Q-domain interaction. For this we selected the Tle-Six3 interaction, which has been shown to be critical for eye development [33], and performed mammalian two-hybrid experiments. Our results showed a stepwise reduction of both Tle1- and Tle4-Six3 complex formation upon increasing Aes concentrations, whereas a control interaction was not affected (Additional file 3). This indicates that Aes overexpression can interfere with both N- and C-terminal interactions of Groucho/Tle proteins in the mixed complexes.

Based on these data we generated two heat-inducible transgenic lines for misexpression of Aes (HSE:Aes) and the Tle4-Q domain (HSE:Q). We then performed heat shock at mid gastrula stage and analysed the embryos over the following days (Fig. 6b-e). In agreement with previous studies we observed eye phenotypes [32, 33]. Interestingly in both transgenic lines a range of phenotypes appeared which might be explained by variations of the heat shock induction and the dependence of the dominant negative approach on the resulting expression levels. We used a phenotype classification scheme similar to the previous one. Embryos with a weak phenotype developed slightly smaller eyes which point towards the midline (Fig. 6c-c”), whereas embryos with a strong phenotype showed cyclopic eyes (Fig. 6e-e”). As a moderate phenotype we considered embryos between these extremes (Fig. 6d-d”). Here, additionally to the weak phenotype, the lenses were shifted anteriorly and mild cyclopia anterior of the forebrain was observed. In HSE:Aes embryos a total of 24% developed a phenotype (*n* = 97), equally distributed between weak and strong. The HSE:Q line showed a total of 34% phenotypes among the induced embryos (*n* = 79), however, only 5% of the embryos were categorized as having a strong phenotype. Therefore, the interference of the Q-domain was less efficient compared to the *aes*-misexpressing line, but interestingly the phenotypes were identical. We then analysed the expression pattern of marker genes for retina (*rx2*), telencephalon (*bf1*) and midbrain (*wnt1*) development in the transgenic embryos at stage 21 (Fig. 6f-k). Supporting the eye phenotypes, the expression of *rx2* was present but restricted to eye size (Fig. 6i). In embryos with a strong phenotype, an additional faint stripe of *rx2* expression that connected the two optic vesicles was observed in the forebrain. In those embryos the expression of *bf1* was also impaired (Fig. 6j). Furthermore, the expression of w*nt1* indicated a slight reduction in the size of the midbrain (Fig. 6k).

In order to elucidate which of the *groucho/tle* genes are involved in eye development we specifically impaired the function of individual genes using antisense oligonucleotides. In medaka, six full length members of the *groucho/tle* family were described; most of them are expressed in largely overlapping domains indicating that they might have redundant functions [34]. We selected three *groucho/tle* paralogs (*tle1, tle2b* and *tle3b*) that are specifically expressed in the optic placode [33, 34]. Interestingly, we observed similar eye phenotypes for all three genes. According to our phenotype classification 10%-16% of the morphants showed eye defects (for more details see Additional file 4), in which the *tle2b*-morphants showed the highest frequencies in strong- (16%, *n* = 237) and eye-less (7%) phenotypes. We next tested combined inactivation and observed an additive effect in *tle1*-*tle2b* morphants (27%, *n* = 44) but not in triple morphants (17%, *n* = 58). The appearance of eye defects in *tle* morphants fully supported our transgenic HSE:aes and HSE:Q results. Nevertheless, we observed some slightly different phenotypes resulting from these two approaches, in particular the cyclopic eye connection in the *tle1* morphants appeared in the middle of the eyes (see Additional file 4C), whereas these connections were preferentially observed anterior to the forebrain in the transgenic HSE:Aes and HSE:Q embryos (Fig. 6d”).

## Discussion

### Knockdown of the tcf3 gene in medaka resembles the zebrafish hdl/tcf3b double morphants

The expression and function of *tcf3* in medaka is poorly understood. In contrast to zebrafish, medaka has only a single *tcf3* gene (this study and [27]), however the expression pattern closely resembled that of the zebrafish *hdl* and *tcf3b* genes (Fig. 1; [19, 20]). It was therefore not surprising that the phenotype of medaka *tcf3* morphants resembled the combined inactivation of both zebrafish *tcf3* paralogs (e.g. complete lack of eyes and anterior brain defects). Knockdown of medaka *tcf3* resulted either in weakly or moderately affected embryos with reduced eye size, covering the range of phenotypes that have been seen for inactivation of single *tcf3* genes in zebrafish, or in strongly affected eye-less embryos corresponding to the complete absence of Tcf3 proteins in zebrafish *hdl/tcf3b* double morphants (Fig. 3). The analysis of marker gene expression in the *tcf3* morphants verifies the strong effect on the Tcf3/Wnt gradient along the AP axis [36, 37], all marker genes tested in this study showed an anterior shift centering on the position of the prechordal plate. These anterior defects are also in good agreement with the AP defects seen in Wnt1 overexpression experiments [38]. Dorsky and colleagues analyzed the expression of these genes in zebrafish upon *tcf3* inactivation. *Hdl* mutants showed a decrease in *pax6* expression in the presumptive head region and *pax2.1* expression was expanded rostrally, whereas the *gbx1* domain remained unchanged. However, if both *hdl* and *tcf3b* were inactivated, *gbx1* also expanded anteriorly, thereby including the expression domain of *pax2.1*, and the anterior expression of *pax6* was completely lost [20]. This stepwise reduction of Tcf3 activity in zebrafish resulted in a progressive anterior shift of the marker genes centring on the position of the prechordal plate [36]. Taken together, a single *tcf3* gene in medaka combines the function of the two *tcf3* paralogous genes in zebrafish.

### Novel side chains provide higher antisense efficiency for PNAs

In this study, we also applied PNAs for *tcf3* knockdown experiments in medaka embryos. We used phosphonic ester modified mixed PNAs, which previously were shown to be suitable for loss-of-function experiments in medaka [9]. Here again we could demonstrate a specific knockdown of gene function. Initially we used pePNAs with two TML end groups and obtained almost 60% specific phenotypes (400 to 900 μM) (Fig. 4a and Additional file 1). At higher concentrations this percentage further increased, however, also unspecific toxicity was observed. The pePNA molecules were also effective in a splice-blocking approach. Although the efficiency was lower compared to the translational blocking experiments, the same *tcf3* specific phenotypes appeared (Fig. 4a and Additional file 1). Furthermore, a combined injection of both PNAs synergistically improved the overall efficiency of the antisense molecules (74% specific phenotypes).

Morpholino oligonucleotides resulted in higher efficiency when directly compared with pePNAs (e.g. 92% and 22% phenotypes at 100 μM, respectively). However, also toxic effects started at considerably lower concentrations (200 μM and 1200 μM, respectively). Otherwise, the obtained phenotypes were identical for the two antisense molecules (Fig. 4b and Additional file 1). Interestingly, further modification of the PNA backbone with lpe-side chains (Fig. 2a) considerably improved the PNA efficiency (Fig. 4b and Additional file 1). At concentrations of 50 μM to 400 μM, 63% to 92% of the embryos showed *tcf3* specific phenotypes (with toxicity first appearing at 600 μM). This efficiency is in the same range as that for the morpholino oligonucleotides. An exchange of 2 nucleotides in this sequence (GT to TG at positions 8 and 9) resulted in a dramatic drop in phenotypes (8% to 16% phenotypes at 100 μM to 400 μM), thus demonstrating the high specificity of this 16mer PNA. Therefore, novel side chain modifications efficiently improved the in vivo antisense function of PNAs.

### Groucho/Tle corepressor proteins are critical for Tcf3 function in eye development

The Tcf3 protein has two major co-repressor interaction domains; an amino-terminally located domain that interacts with Groucho/Tle proteins [24] and a carboxy-terminal domain that interacts with the CtBP protein [29]. Our results indicate that the CtBP interacting domain in Tcf3 is dispensable for early embryonic eye development; indicating that the interaction with Groucho/Tle proteins is more crucial. In particular, Tcf3 variants lacking the Groucho/Tle interaction domain showed considerably reduced numbers of eye phenotypes in our gain-of-function experiments. These results further support the idea that the Groucho/Tle co-repressors are required for eye development [33, 39].

To interfere with the function of all Groucho/Tle proteins we used three strategies. Firstly, we misexpressed the *aes* gene. Aes (Fig. 6) is a naturally occurring truncated family member of full-length Groucho/Tle proteins that consists of only the Q and the GP domains [25, 26, 40]. The Q domain allows Aes to oligomerize with full-length Groucho/Tle proteins, thereby reducing the number of C-terminal interaction domains [21, 33, 41–45]. The GP domain, however, is functionally different from full-length Groucho/Tle proteins since it does not interact with HDAC co-repressors [13, 39, 46, 47]. It was therefore suggested that through this interaction Aes turns Groucho/Tle oligomers into non-functional complexes, which therefore have a reduced number of C-terminal WD-40 interaction domains and a reduced efficiency to cooperate with HDAC co-repressors [21, 41, 42, 44, 48, 49]. Supportive of this hypothesis, our mammalian two-hybrid experiments using the Six3 transcription factor, indicated that additionally N-terminal interactions with the Q-domain are blocked by Aes misexpression. Indeed, ectopic expression of Aes in medaka resulted in considerably smaller eyes and otic vesicles and reduced expression of optic and otic marker genes, suggesting an efficient block of Groucho/Tle activity [32, 33]. In addition to the Groucho/Tle blocking functions, Aes is also known to exhibit specific functions on its own in certain cellular contexts [46, 50, 51]. The observed phenotypes could therefore be a mixture between *groucho/tle* loss-of-function and *aes* gain-of-function effects. Secondly, we misexpressed the Tle4 Q-domain and could phenocopy the Aes misexpression embryos, however, at a lower efficiency. The reduction of the *bf1* and *wnt1* expression field in the forebrain and midbrain in those embryos also confirmed the observed phenotypes and is in agreement with a previous study in *Xenopus* [52]. Finally, we analysed the function of *groucho/tle* genes by injection of morpholino oligonucleotides. Previously, we have shown that three paralogs of medaka *groucho/tle* genes (*tle1*, *tle2b* and *tle3b*) are expressed during early eye development [34]. Interestingly, knockdown of each of these genes, individually or in combination, resulted in specific impairment of eye development which suggests redundancy. These phenotypes closely resembled those of the dominant negative experiments and thus fully support this approach.

In addition to eye phenotypes, we also observed ectopic otic vesicles in the Tcf3[1-434]ΔGro- injected embryos. The appearance of ectopic otic vesicles might be caused by a dominant negative effect of the overexpressed truncated Tcf3, thus blocking the repressing function of full-length Tcf3. Such an anti-repressive effect would be equivalent to ectopic Wnt activation. Indeed, we have previously shown that induction of Wnt signaling leads to the formation of ectopic otic vesicles [38, 53].

## Conclusion

Taken together, our results suggest that a single *tcf3* gene in medaka is crucial for eye development and its function is modulated by interaction with Groucho/Tle co-repressor proteins. Gain-of-function analyses of Aes and the Q-domain, as dominant negative effectors, together with the loss-of-function analyses of individual *tle* genes confirm this interpretation. Moreover, our results indicate that three *tle* genes (*tle1, tle2b* and *tle3b*) might have redundant functions during eye development.

## Methods

### PNA and morpholino oligonucleotide synthesis

Lysine-phosphonic-ester Ugi-PNA monomers were synthesized according to the following process. First, an N-Cbz or N-Boc protected phosphono glycinate was reacted with the appropriate aldehyde in a Horner- Wadsworth-Emmons [54–56] followed by enantioselective hydrogenation using Burk’s catalyst [57–59] to give the α-substituted protected glycinate. Deprotection of the α-amino function and subsequent reductive amination with N-Boc-amino acetaldehyde gave the Ugi-PNA-monomer backbone. The latter was coupled to the appropriate protected nucleobase acetic acid component, employing HATU as coupling reagent. Finally, deprotection of the carboxylic function yielded the Ugi PNA monomer. Synthesis of the C3-phosphonic-ester-Ugi-PNA monomer building blocks was performed as described previously [9]. The PNAs were dissolved to 1 mM in nuclease free water by repeated shaking and vortexting. Finally they were gently sonicated for 2 min with repeated pulses. Subsequently the PNAs were kept at −80 °C. Phosphorodiamidate morpholino oligonucleotides (abbreviated here as morpholinos) were manufactured by Gene Tools (see Fig. 3). Morpholino sequences for the *tle* genes in 5′-3′ direction: *tle1*, CGCGTCTTGTCCTGAAACCCCGCTA; *tle2b*, GGCAGTGCGTCCTCGTGGCTCTTTC; *tle3b*, CGGCCTTGTGGATACATGTCTCGTC. For injection into medaka embryos, morpholinos were dissolved in 1× Yamamoto’s Ringer solution at the indicated concentration and injected into the blastomere at the one-cell stage.

### DNA constructs

All DNA constructs for microinjection were under the control of a bi-directional heat-inducible promoter [28] and contained either the mouse Aes/Grg5 cDNA, the mouse Tle4 Q domain (AA 1-135), or various deletion fragments of Tcf3 cDNA (see Fig. 5 for details). For cell culture experiments pMC [60] and pKC (polylinker modification of pKW) [61] were used (both vectors contain cytomegalovirus promoters). pLucF24ZF; luciferase reporter under the control of a Fos minimal promoter and ZFHD binding sites. pMChSix3(85-203)mZFb6; the human Six3 fragment (positions of amino acids in brackets) fused to a ZFHD DNA binding domain [62] and the “base” leucine-zipper [63]. pMCTle1VP16 and pMCTle4VP16; human Tle1 and mouse Tle4 were fused to the transcriptional activation domain of the herpes simplex virus protein VP16, respectively. pMCacidVP16 contains the “acid” leucine-zipper fused to VP16, pKCAes the mouse Aes/Grg5 cDNA.

### Microinjection into medaka embryos

Embryos of the medaka Cab strain were used for all experiments. Adult fish were maintained at 26 °C with an artificial 14 h light and 10 h dark cycle. Stages were determined according to the morphological markers described in Iwamatsu [64]. DNA constructs were co-injected with I-SceI meganuclease enzyme (0.5 U/μl) at the one-cell stage. For transient studies, different versions of truncated *tcf3* cDNAs (HSE:*tcf3* constructs) were injected at a concentration of 40 ng/μl. For the generation of a heat-inducible *aes* (HSE:*aes*) and Q-domain (HSE:Q) transgenic lines, DNA constructs were injected at 10 ng/μl concentration together with I-SceI meganuclease enzyme. The embryos were incubated at normal conditions (1× ERM buffer at 27 °C). Heat treatment was performed at stage 14 (except if otherwise indicated) for 10 min at 43.5 °C. For co-injection experiments FITC-dextran (FD10S, Sigma-Aldrich) was used.

### Whole-mount in situ hybridization

Whole-mount in situ hybridization using DIG-labelled RNA probes was performed as described previously [34]. Probes against *tcf3* (Ensembl: ENSORLT00000014810) and *gbx1* (Ensembl: ENSORLT00000021915) were PCR amplified from medaka embryonic cDNA using specific primers for *tcf3* (5’-CGGATCCATGGCTCAACTGAACGGAGGC-3′ and 5’-CGAAGACGGCTGGACATGGATGCATTCA-3′) and *gbx1* (5’-TTAGAAAATACAGCCACAA-3′ and 5’-TCACTGTAAAAAGTACCTG-3′). The expression patterns of *pax6* [65], *pax2* [66], *wnt1* [67], *rx2* [30] and *bf1* [68] were described previously.

### Cell culture and mammalian two-hybrid assay

HeLa cells (300,194, Cell Line Services) were transfected in polyethylenimine (2.5 μg/ml) coated 96-well plates [69] using TurboFect transfection reagent according to the instructions of the manufacturer (Thermo Scientific). For luciferase activity detection, additional transfection of 2 ng Gaussia luciferase expression vector pMCGlucS served as an internal reference. Luciferase activities were measured 24 h after transfection. For the mammalian two-hybrid assay, cells were co-transfected with 70 ng of firefly luciferase reporter construct pLucF24ZF, 20 ng of Six3 expressing plasmid pMChSix3(85-203)mZFbase (six-domain of human Six3), 0-6 ng of Aes expressing plasmid pKCAes, and 20 ng of either pMCTle1VP16, Tle4 pMCTle4VP16 or the control pMCacidVP16.

## Additional file

Additional file 1: Overview of the PNA and MO induced loss of function phenotypes. Embryos at the 1-cell stage were injected with PNAs or MOs at the indicated concentrations. Phenotypes were categorized according to the size of the eyes into: weak, slightly smaller eyes; moderate, smaller eyes; strong, eye-less. Abbreviations: PNA, peptide nucleic acid; MO, morpholino oligonucleotides. (PDF 10028 kb)
Additional file 2: Gro/Tle dependence of 1 *tcf3* in gain-of-function experiments. Embryos at the 1-2 cell stage were co-injected with 40 ng/μl of the indicated gfp:HSE:Tcf3 constructs. Heat treatment (10 min, 43.5 °C) was applied at stage 14. (A) Statistical overview of the phenotype distribution. Whole mount in situ hybridization experiments for rx2 were performed on embryos at stage 21. (B) dorsal view of a stage 31 embryo with anterior at the top, (C) lateral view with anterior at the left. Arrowheads indicate ectopic otic vesicles, the arrow points to the endogenous otic vesicle. Scale bar 100 μm; B and C. (PDF 10028 kb)
Additional file 3: Mammalian two-hybrid analysis of Aes-mediated Gro/Tle repression. 20 ng of the expression constructs (pMCamVP16, pMCTle4VP16 or pMCTle1VP16) were co-transfected with 70 ng of firefly luciferase reporter construct pLucF24ZF, 20 ng of the expression construct for the six-domain of human Six3 (pMChSix3(85-203)mZFb6) and full length Aes (pKCAes at the indicated concentrations) into HeLa cells. The interaction of the artificial leucine zipper domain “acid” and “base” [63] was used as a control. All three interactions were set 100% in the absence of Aes (acid/base corresponds to a more than 100 fold activation of the luc reporter construct compared to a control without bait, similar induction rates were seen for Tle4/Six3 and Tle1/Six3). Addition of the Aes expression construct at the indicated concentrations did not affect the acid/base interaction (blue bars), but strongly reduced the Tle4/Six3- (red bars) and the Tle1/Six3 interaction (green bars). (PDF 10028 kb)
Additional file 4: MO-induced *gro/tle* loss of function phenotypes. Embryos at the 1-cell stage were co-injected with morpholino oligonucleotides and 1 μg/ml FITC-dextran. Injections were performed using either a single morpholino directed against *tle1* (C), *tle2b* (D,F), and *tle3b* (E), or combinations directed against *tle1 + 2b* (H), *tle1 + 3b* (I), *tle2b + 3b* (J), and *tle1 + 2b + 3b* (K). Single morpholino oligonucleotides were injected at a concentration of 600 μM (C-F) and combinatorial injections (H-K) were performed using 300 μM of each MO. Phenotypes of FITC-dextran positive embryos were observed after the beginning of eye pigmentation at stage 32 (B-F) and stage 28 (G-K). (B,G) Wild type control embryos were injected with 1× Yamamoto’s and FITC-dextran. All embryos are shown in dorsal view with anterior at the top. Compared to the wild type controls (B,G), morpholino injected embryos developed smaller eyes that were shifted towards the midline (D,E,J,K) or cyclopic eyes (C,H,I). In rare cases the eyes were lost entirely (E). (A) Phenotypes of FITC-positive embryos were categorized at stage 28-32 into weak and strong phenotypes. Weak phenotypes developed smaller eyes that were shifted towards the midline, whereas strong phenotypes showed cyclopic eyes. Eye-less phenotypes were included into the group of strong phenotypes. Abbreviations: MO, morpholino oligonucleotide; WT, wild type. Scale bar 100 μM. (PDF 10028 kb)

## Abbreviations

aegPNA: N-[2-aminoethyl]-glycine PNA
AP: Anterior-posterior
lpe: Lysine-phosphonic-ester
MHB: Mid-hindbrain boundary
MO: Morpholino oligonucleotide
pePNA: Phosphonic ester PNA
PNA: Peptide Nucleic Acid
TML: Tri-methyl-lysine
