# Supplementary material for: The function of tcf3 in medaka embryos: efficient knockdown with pePNAs
Source: BMC Biotechnol. 2018 Jan 9;18:1. doi: 10.1186/s12896-017-0411-0 (PMC5759164; doi:10.1186/s12896-017-0411-0)
Supplement: Additional file 1: — Overview of the PNA and MO induced loss of function phenotypes. Embryos at the 1-cell stage were injected with PNAs or MOs at the indicated concentrations. Phenotypes were categorized according to the size of the eyes into: weak, slightly smaller eyes; moderate, smaller eyes; strong, eye-less. Abbreviations: PNA, peptide nucleic acid; MO, morpholino oligonucleotides. (PDF 10028 kb) [file 12896_2017_411_MOESM1_ESM.pdf]

Additional File 1

	concentration [µM]	0	50	100	200	400	600	900	1200	200+200	300+300	400+400	600+600
Tcf3PNA	number of embryos	17		72	72	110	123	52	90				
	dead	1		5	2	10	8	3	34				
	death rate	6%		7%	3%	9%	7%	6%	38%				
	strong phenotype	0		0	2	10	15	3	24				
	moderate phenotype	0		3	7	12	17	10	12				
	weak phenotype	0		12	18	29	33	15	12				
	normal	16		52	43	49	50	21	8				
	phenotypes in surviv.	0%		22%	39%	51%	57%	57%	86%				
Tcf3spPNA	number of embryos	45		32	34	59	101	83	55				
	dead	2		2	2	3	21	7	49				
	death rate	4%		6%	6%	5%	21%	8%	89%				
	strong phenotype	0		0	0	2	11	6	1				
	moderate phenotype	0		0	1	5	8	11	1				
	weak phenotype	0		2	2	10	20	18	3				
	normal	43		28	29	39	41	41	1				
	phenotypes in surviv.	0%		7%	9%	30%	49%	46%	83%				
Tcf3PNA+Tcf3spPNA	number of embryos	37								45	93	121	42
	dead	1								1	15	17	30
	death rate	3%								2%	16%	14%	71%
	strong phenotype	0								2	20	24	2
	moderate phenotype	0								7	18	19	0
	weak phenotype	0								13	14	34	9
	normal	36								22	26	27	1
	phenotypes in surviv.	0%								50%	67%	74%	92%
Tcf3kPNA	number of embryos	19	47	48	45	92	78	42					
	dead	3	7	7	8	18	33	30					
	death rate	16%	15%	15%	18%	20%	42%	71%					
	strong phenotype	0	15	6	6	26	21	7					
	moderate phenotype	0	6	6	11	26	10	0					
	weak phenotype	0	4	19	12	16	11	0					
	normal	16	15	10	8	6	3	5					
	phenotypes in surviv.	0%	63%	76%	78%	92%	93%	58%					
Tcf3kPNAmut	number of embryos	25		24	46	49	53						
	dead	3		2	7	8	17						
	death rate	12%		8%	15%	16%	32%						
	strong phenotype	0		0	0	1	0						
	moderate phenotype	0		0	0	1	1						
	weak phenotype	0		0	2	3	3						
	normal	22		22	37	36	32						
	phenotypes in surviv.	0%		0%	5%	12%	11%						
Tcf3MO	number of embryos	9	171	112	34								
	dead	1	17	14	15								
	death rate	11%	10%	13%	44%								
	strong phenotype	0	65	66	16								
	moderate phenotype	0	19	19	2								
	weak phenotype	0	25	5	1								
	normal	8	45	8	0								
	phenotypes in surviv.	0%	71%	92%	100%								

Additional File 1. Overview of the PNA and MO induced loss of function phenotypes. Embryos at the 1-cell stage were injected with PNAs or MOs at the indicated concentrations. Phenotypes were categorized according to the size of the eyes into: weak, slightly smaller eyes; moderate, smaller eyes; strong, eye-less. Abbreviations: PNA, peptide nucleic acid; MO, morpholino oligonucleotides.
