# Supplementary material for: The function of tcf3 in medaka embryos: efficient knockdown with pePNAs
Source: BMC Biotechnol. 2018 Jan 9;18:1. doi: 10.1186/s12896-017-0411-0 (PMC5759164; doi:10.1186/s12896-017-0411-0)
Supplement: Additional file 2: — Gro/Tle dependence of 1 tcf3 in gain-of-function experiments. Embryos at the 1-2 cell stage were co-injected with 40 ng/μl of the indicated gfp:HSE:Tcf3 constructs. Heat treatment (10 min, 43.5 °C) was applied at stage 14. (A) Statistical overview of the phenotype distribution. Whole mount in situ hybridization experiments for rx2 were performed on embryos at stage 21. (B) dorsal view of a stage 31 embryo with anterior at the top, (C) lateral view with anterior at the left. Arrowheads indicate ectopic otic vesicles, the arrow points to the endogenous otic vesicle. Scale bar 100 μm; B and C. (PDF 10028 kb) [file 12896_2017_411_MOESM2_ESM.pdf]

Additional File 2

A

	rx2	
	**Tcf3(1-434)**	**Tcf3(1-434)∆Gro**
Embryos	10	10
Weak phenotypes	1	4
Moderate phenotypes	5	1
Strong phenotypes	3	
Total phenotypes	90%	50%

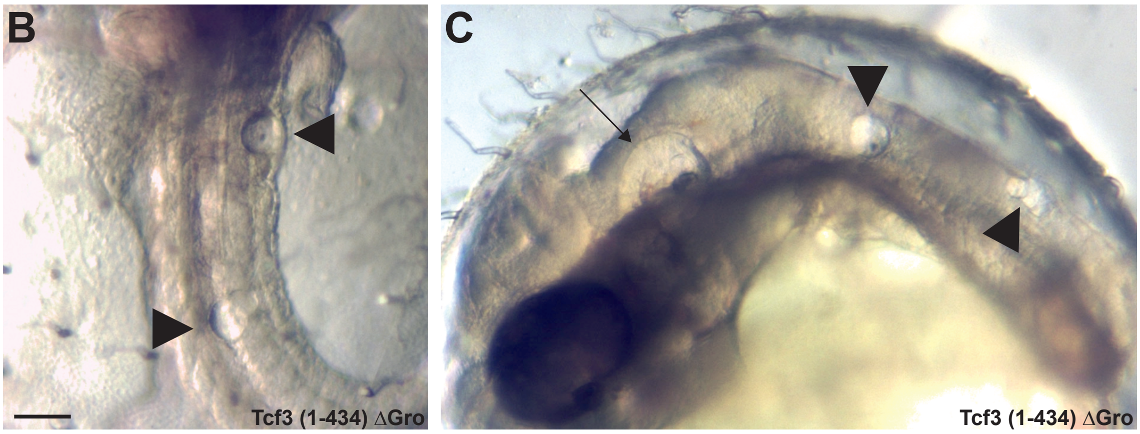

Additional File 2. Gro/Tle dependence of *tcf3* in gain-of-function experiments. Embryos at the 1-cell stage were co-injected with 40 ng/µl of the indicated gfp:HSE:Tcf3 constructs. Heat treatment (10 min, 43.5°C) was applied at stage 14. (A) Statistical overview of the phenotype distribution. Whole mount *in situ* hybridization experiments for *rx2* were performed on embryos at stage 21. (B) dorsal view of a stage 31 embryo with anterior at the top, (C) lateral view with anterior at the left. Arrowheads indicate ectopic otic vesicles, the arrow points to the endogenous otic vesicle. Scale bar 100 µm; B, B and C.
