# Supplementary material for: The function of tcf3 in medaka embryos: efficient knockdown with pePNAs
Source: BMC Biotechnol. 2018 Jan 9;18:1. doi: 10.1186/s12896-017-0411-0 (PMC5759164; doi:10.1186/s12896-017-0411-0)
Supplement: Additional file 3: — Mammalian two-hybrid analysis of Aes-mediated Gro/Tle repression. 20 ng of the expression constructs (pMCamVP16, pMCTle4VP16 or pMCTle1VP16) were co-transfected with 70 ng of firefly luciferase reporter construct pLucF24ZF, 20 ng of the expression construct for the six-domain of human Six3 (pMChSix3(85-203)mZFb6) and full length Aes (pKCAes at the indicated concentrations) into HeLa cells. The interaction of the artificial leucine zipper domain “acid” and “base” [63] was used as a control. All three interactions were set 100% in the absence of Aes (acid/base corresponds to a more than 100 fold activation of the luc reporter construct compared to a control without bait, similar induction rates were seen for Tle4/Six3 and Tle1/Six3). Addition of the Aes expression construct at the indicated concentrations did not affect the acid/base interaction (blue bars), but strongly reduced the Tle4/Six3- (red bars) and the Tle1/Six3 interaction (green bars). (PDF 10028 kb) [file 12896_2017_411_MOESM3_ESM.pdf]

Additional File 3

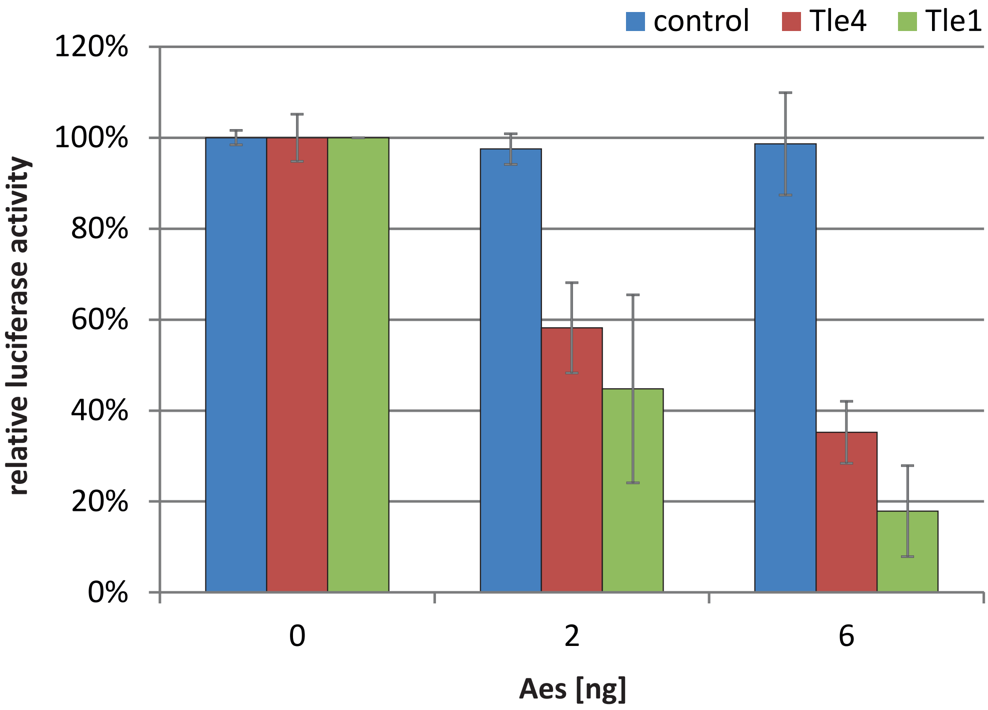

Additional File 3. Mammalian two-hybrid analysis of Aes-mediated Gro/Tle repression. 20 ng of the expression constructs (pMCamVP16, pMCTle4VP16 or pMCTle1VP16) were co-transfected with 70 ng of firefly luciferase reporter construct pLucF24ZF, 20 ng of the expression construct for the six-domain of human Six3 (pMChSix3(85-203)mZFb6) and full length Aes (pKCAes at the indicated concentrations) into HeLa cells. The interaction of the artificial leucine zipper domain "acid" and "base" [63] was used as a control. All three interactions were set 100% in the absence of Aes (acid/base corresponds to a more than 100 fold activation of the luc reporter construct compared to a control without bait, similar induction rates were seen for Tle4/Six3 and Tle1/Six3). Addition of the Aes expression construct at the indicated concentrations did not affect the acid/base interaction (blue bars), but strongly reduced the Tle4/Six3- (red bars) and the Tle1/Six3 interaction (green bars).
