# Supplementary material for: The function of tcf3 in medaka embryos: efficient knockdown with pePNAs
Source: BMC Biotechnol. 2018 Jan 9;18:1. doi: 10.1186/s12896-017-0411-0 (PMC5759164; doi:10.1186/s12896-017-0411-0)
Supplement: Additional file 4: — MO-induced gro/tle loss of function phenotypes. Embryos at the 1-cell stage were co-injected with morpholino oligonucleotides and 1 μg/ml FITC-dextran. Injections were performed using either a single morpholino directed against tle1 (C), tle2b (D,F), and tle3b (E), or combinations directed against tle1 + 2b (H), tle1 + 3b (I), tle2b + 3b (J), and tle1 + 2b + 3b (K). Single morpholino oligonucleotides were injected at a concentration of 600 μM (C-F) and combinatorial injections (H-K) were performed using 300 μM of each MO. Phenotypes of FITC-dextran positive embryos were observed after the beginning of eye pigmentation at stage 32 (B-F) and stage 28 (G-K). (B,G) Wild type control embryos were injected with 1× Yamamoto’s and FITC-dextran. All embryos are shown in dorsal view with anterior at the top. Compared to the wild type controls (B,G), morpholino injected embryos developed smaller eyes that were shifted towards the midline (D,E,J,K) or cyclopic eyes (C,H,I). In rare cases the eyes were lost entirely (E). (A) Phenotypes of FITC-positive embryos were categorized at stage 28-32 into weak and strong phenotypes. Weak phenotypes developed smaller eyes that were shifted towards the midline, whereas strong phenotypes showed cyclopic eyes. Eye-less phenotypes were included into the group of strong phenotypes. Abbreviations: MO, morpholino oligonucleotide; WT, wild type. Scale bar 100 μM. (PDF 10028 kb) [file 12896_2017_411_MOESM4_ESM.pdf]

Additional File 4

A

Morpholino<br>Concentration	*tle 1*<br>600 µM	*tle 2b*<br>600 µM	*tle 3b*<br>600 µM	*tle 1+2b*<br>300 µM each	*tle 1+3b*<br>300 µM each	*tle 2b+3b*<br>300 µM each	*tle 1+2b+3b*<br>300 µM each
Embryos	95	267	117	55	115	45	58
Dead	11	30	6	11	16	5	4
Mortality	12%	11%	5%	20%	14%	11%	7%
Weak	4	12	1	7	16	3	1
Moderate	2	11	10	0	1	3	5
Strong	2	16	2	5	4	0	3
Eye-less*)	0	7	1	3	3	0	0
Eye phenotypes in surviving embryos	10%	16%	12%	27%	21%	15%	17%

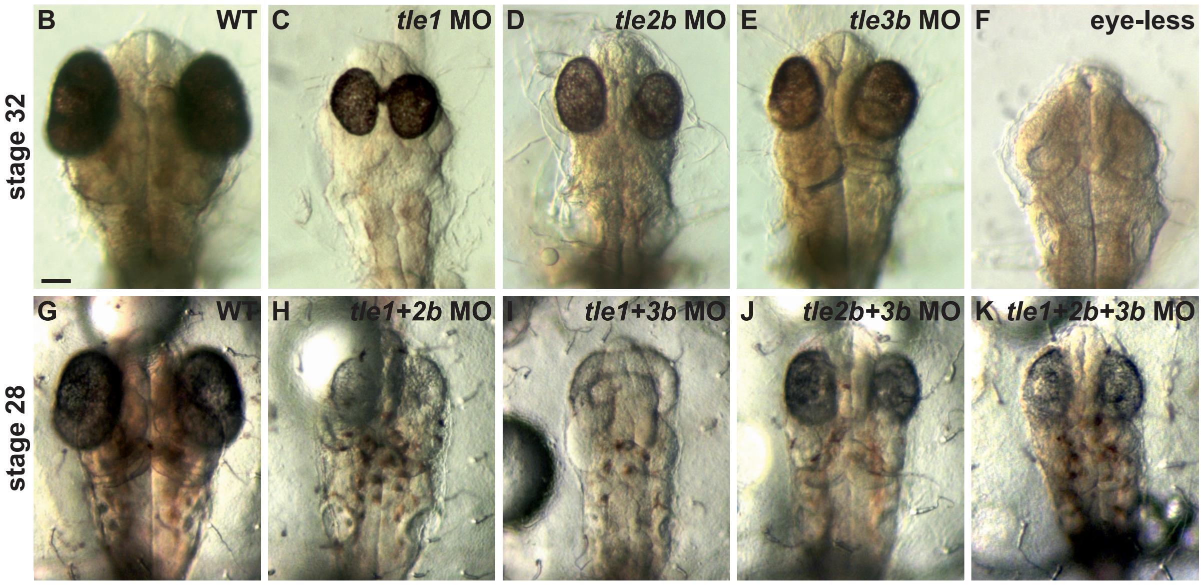

Additional File 4. MO-induced *gro/tle* loss of function phenotypes. Embryos at the 1-cell stage were co-injected with morpholino oligonucleotides and 1 µg/ml FITC-dextran. Injections were performed using either a single morpholino directed against *tle1* (C), *tle2b* (D,F), and *tle3b* (E), or combinations directed against *tle1+2b* (H), *tle1+3b* (I), *tle2b+3b* (J), and *tle1+2b+3b* (K). Single morpholino oligonucleotides were injected at a concentration of 600 µM (C-F) and combinatorial injections (H-K) were performed using 300 µM of each MO. Phenotypes of FITC-dextran positive embryos were observed after the beginning of eye pigmentation at stage 32 (B-F) and stage 28 (G-K). (B,G) Wild type control embryos were injected with 1x Yamamoto's and FITC-dextran. All embryos are shown in dorsal view with anterior at the top. Compared to the wild type controls (B,G), morpholino injected embryos developed smaller eyes that were shifted towards the midline (D,E,J,K) or cyclopic eyes (C,H,I). In rare cases the eyes were lost entirely (E). (A) Phenotypes of FITC-positive embryos were categorized at stage 28-32 into weak and strong phenotypes. Weak phenotypes developed smaller eyes that were shifted towards the midline, whereas strong phenotypes showed cyclopic eyes. Eye-less phenotypes were included into the group of strong phenotypes. Abbreviations: MO, morpholino oligonucleotide; WT, wild type. Scale bar 100 µM.
